# Aerobic Commensal Conjunctival Microflora in Healthy Donkeys

**DOI:** 10.3390/ani12060756

**Published:** 2022-03-17

**Authors:** Kaja Fraczkowska, Agnieszka Zak-Bochenek, Natalia Siwinska, Krzysztof Rypula, Katarzyna Ploneczka-Janeczko

**Affiliations:** 1 Faculty of Veterinary Medicine, Wroclaw University of Environmental and Life Sciences, 50-375 Wroclaw, Poland; kaja.fraczkowska@gmail.com; 2 Department of Immunology, Pathophysiology and Veterinary Preventive Medicine, Faculty of Veterinary Medicine, Pathophysiology and Veterinary Preventive Medicine, Wrocław University of Environmental and Life Sciences, 50-375 Wroclaw, Poland; 3 Department of Internal Diseases and Clinic of Diseases of Horses, Dogs and Cats, Faculty of Veterinary Medicine, Pathophysiology and Veterinary Preventive Medicine, Wrocław University of Environmental and Life Sciences, 50-375 Wroclaw, Poland; natalia.siwinska@upwr.edu.pl; 4 Department of Epizootiology and Clinic for Birds and Exotic Animals, Faculty of Veterinary Medicine, Pathophysiology and Veterinary Preventive Medicine, Wrocław University of Environmental and Life Sciences, 50-375 Wroclaw, Poland; krzysztof.rypula@upwr.edu.pl (K.R.); katarzyna.ploneczka-janeczko@upwr.edu.pl (K.P.-J.)

**Keywords:** donkeys, conjunctival flora, commensals, Central Europe

## Abstract

**Simple Summary:**

The mucosa of the conjunctival sac in mammals is colonized by a group of bacteria, and their interdependence is referred to as commensalism—the coexistence of two different species populations benefiting at least one of the partners. The presence of conjunctival bacterial commensal flora is very important for the capacity of the non-specific immune response. Some of the bacteria are described as conditionally pathogenic, meaning that in the case of breaking down protective barriers, they may act as pathogens. Knowledge of the commensal flora of healthy individuals is of great clinical importance in the diagnosis of pathological conditions. The aim of this study is to assess the presence and the composition of normal ocular microflora of healthy donkeys. To the best of the authors’ knowledge, this is the first report that provides knowledge about commensal microflora in the conjunctival sac of donkeys bred in Central Europe.

**Abstract:**

From a clinical point of view, knowledge of the commensal microbial flora of the conjunctival sac in healthy individuals proves to be of great importance. The aim of this study was to assess the presence and the composition of normal ocular microflora of healthy donkeys. Fourteen clinically healthy donkeys (*Equus asinus*) participated in the study. After prior ophthalmological examination, which showed no abnormalities, a conjunctival swab was taken from each donkey from the right and left eye. Species-specific identification was based on a morphological assessment of bacterial colonies stained with the Gram technique, as well as on biochemical properties and the disk-diffusion method. Around 82% of samples were positive for bacteria cultivation; *Pantoea agglomerans* was the most prevalently detected species, followed by *Moraxella lacunata*. In conclusion, our study made it possible to determine the commensal flora of the conjunctival sac in donkeys. The obtained results also showed discrepancies in the composition of the conjunctival sac flora of donkeys and horses, despite the geographical proximity of performed studies. Knowledge of the commensal conjunctival flora of donkeys is of great clinical importance due to their greater exposure to corneal damage and infections than horses.

## 1. Introduction

It is known that the composition of the microflora on the surface of a healthy eye is controlled by a number of innate and acquired immune mechanisms that maintain the balance between microbial populations, preventing the overgrowth of any species. These mechanisms include: the barrier function of the epithelium, mucins, the tear film flushing out impurities from the eye, tear’s components with antimicrobial activity (such as lactoferrin, lysozyme, beta-lysine, immunoglobulin A, leukocytes), antigen-presenting cells and Toll-like receptors recognizing antigens [1,2,3,4]. The commensal flora is also an important protective factor against pathogenic microbes by limiting the surface area available for the growth of other microorganisms, consuming the nutrient components on the epithelial surface and the production of antimicrobial substances [4,5].

In pathological conditions, such as a corneal abrasion, when defense mechanisms are broken, commensal microorganisms may play the role of opportunistic pathogens. Bacteria considered earlier as commensals can cause keratitis or an infected corneal ulcer that is difficult to treat and can lead to blindness. It has been reported that Gram-negative bacteria are isolated more frequently than Gram-positive bacteria from the cases of ulcerative keratitis in horses [6].

There are numerous studies describing the bacterial flora of horses’ conjunctiva, but only one investigating the normal conjunctival bacterial flora of donkeys [4,7,8,9]. Another study presents the conjunctival fungal flora of healthy donkeys in Italy [10]. Most research of the normal conjunctival microflora in horses showed a predominance of commensal, mainly Gram-positive bacteria. Nevertheless, Gram-negative bacteria have also been found in the conjunctiva of clinically healthy horses [8,11,12]. A similar relationship between Gram-positive and Gram-negative bacteria was also observed in the conjunctiva of donkeys in Italy [7]. However, due to the scarcity of research on donkeys, this species should be investigated more, taking into account geographic, environmental and maintenance differences. From an epidemiological point of view, the commensal flora of both animals and humans can mix between these species as well.

The aim of this study was to assess the presence and the composition of normal ocular microflora of healthy donkeys. To the best of the authors’ knowledge, this is the first report that provides knowledge about commensal microflora in the conjunctival sac of donkeys bred in Central Europe.

## 2. Material and Methods

### 2.1. Sampling the Ocular Swabs

Fourteen donkeys (*Equus asinus*) of mix-breed and both sexes participated in the study. In the examined group, there were: mares, stallions and geldings, with a mean age of 8.4 years (range 2 to 16). All animals were kept within one herd in a breeding center in Poland. The animals had constant access to pasture, fresh water and mineral licks. They were additionally fed with hay. All animals were vaccinated against influenza and tetanus and dewormed according to the preventive program. Oral consent of the owner was obtained prior to sample collection. According to the present law in Poland (the Experiments on Animals Act from 15 January 2015, Journal of Laws of the Republic of Poland from 2015, item. 266), the study did not require the approval of the Ethics Committee. The general health status of animals was assessed, and the basal clinical examination showed a lack of abnormalities, such as nasal or ocular discharge, skin maceration, cough, fever, etc. The owners declared no ophthalmic problems in the last two months prior to sampling. A screening ophthalmological examination was carried out using a source of light and a direct ophthalmoscope (Ri-scope L2, Riester, Jungingen, Germany). Due to the described potential bacterial effect of fluorescein, no staining of the cornea was performed [12]. A total of 28 conjunctival swabs, taken from both conjunctival sacs from 14 healthy donkeys, were examined in 2019. The swabs were collected once from both eyes (Right and Left) of each donkey without sedation or local anesthesia, as has been previously described [12]. In order to take an eye swab, the conjunctiva of the lower and third eyelid was visualized by retropulsion of the eye globe [12]. The sample was collected using a cotton-tipped sterile swab, covered by a sterile saline swab (Sarstedt, Copan, Italia) from the conjunctival sac [12]. The procedure was carried out carefully so as not to contaminate the sample through contact with whiskers or skin. After sampling, the swabs were placed into the transport container and transferred for microbiological analysis into the diagnostic laboratory within an hour at room temperature (24 °C). The animals were observed daily for the next two weeks, and no ophthalmological problems were found.

### 2.2. Microbiological Analysis

The microbiological analysis was carried out using established techniques at the “Epi-Vet”, a veterinary diagnostic laboratory at the Department of Epizootiology with the Clinic for Birds and Exotic Animals of the Wroclaw University of Environmental and Life Sciences, in accordance with the prevailing microbiological regulations (according to ISO 9001:2008) and the Manual of Clinical Microbiology [13]. Collected swabs were transported on the semi-solid Amies transport medium (Meus S.r.l., Arzergrande, Italy), a commercial transport medium for clinical samples, for aerobic and anaerobic bacteria. After transfer to the laboratory, specimens were planted onto Chapman medium (Mannitol Salt Agar, MSA; Oxoid Ltd., Basingstoke, UK), Columbia Agar (enriched blood agar with 5% addition of sheep blood; Oxoid Ltd., Basingstoke, UK), Chromogenic medium (Chromogenic UTI Clarity Agar, Oxoid Ltd., Basingstoke, UK) and Saboraud Agar with chloramphenicol and gentamicin (Oxoid Ltd., Basingstoke, UK) presented below. All commercial media were purchased regularly by the “Epi-Vet” Laboratory under a tender procedure. The plates were then incubated at 37 °C for 24–48 h under aerobic conditions. The tubes with swabs were incubated in the same condition overnight. Plates were examined at 24 h. If no growth was evident, the subcultures from incubated tubes with swabs were planted onto fresh growth media and incubated in the same conditions as previously. The culture was reported as negative if no growth was observed within 48 h.

### 2.3. Species Strain Identification

The preliminary identification of isolates was performed according to the standard microbiological protocols based on growth on the different media, the bacterial or fungi colony morphology (appearance, size, texture, fragrance, the presence of spores), Gram staining, as well as on biochemical properties and antibiotic-susceptibility testing by Kirby–Bauer method.

Prior to the evaluation of the biochemical properties, a pure bacterial culture of examined microorganisms was cultivated (24–48 h, 37 °C). In the disc diffusion method, according to the guidelines of the Clinical & Laboratory Standards Institute (CLSI), all determinations were carried out on Mueller–Hinton agar (Liofilchem, Teramo, Italy) [14]. Agar plates were inoculated with a standardized inoculum (0.5 g in the Mc Farland’s scale) of the examined microorganisms, and then the discs with Novobiocin 5 µg and Polymyxin B 300 µg (NV 5, BP 300, Oxoid Ltd., Basingstoke, UK) were placed on the agar surface. After pre-incubation (20 and 30 min respectively; room temperature) and overnight incubation of the plates at 37 °C, the diameters of inhibition growth zones were measured. Isolates were classified as resistant to Novobiocin and Polymyxin B when the inhibition growth zones were ≤12 mm. Similarly, the susceptibility of the *Staphylococcus* spp. isolates to methicillin were examined on the Mueller–Hinton agar using 30 μg Cefoxitin discs (FOX 30 Cefoxitin, Oxoid Ltd., Basingstoke, UK), with incubation conditions as described above [15]. All commercial tests, such as ENTEROtest 16 (Erba Lachema S.r.o., Brno, Czech Republic), RapID^TM^ STAPH PLUS (Thermo Fisher SCIENTIFIC., Waltham, MA, USA) and RapID^TM^ NF PLUS System (Thermo Fischer SCIENTIFIC, Waltham, MA, USA), were carried out in accordance with the manufacturer’s instruction. In order to identify the species, the pure bacterial colonies were examined using the following commercial tests: the oxidase test (Oxidase Reagent, Liofilchem^®^, Teramo, Italy); for oxidase-negative bacteria (no color change) and for oxidase-positive bacteria (purple color), ENTEROtest 16 and RapID^TM^ NF PLUS System were used respectively; the catalase test (Catalase Reagent, solution of 3% hydrogen peroxide, Liofilchem^®^, Italy); the coagulase test (Coagulase Plasma, Oxoid Ltd., Basingstoke, UK); Staphaurex^TM^ Plus Agglutination Test (Oxoid Ltd., Basingstoke, UK); RapID^TM^ STAPH PLUS System. At the satisfactory probability level (>99.9%), species-specific identification was estimated.

## 3. Results

**Figure 1:**
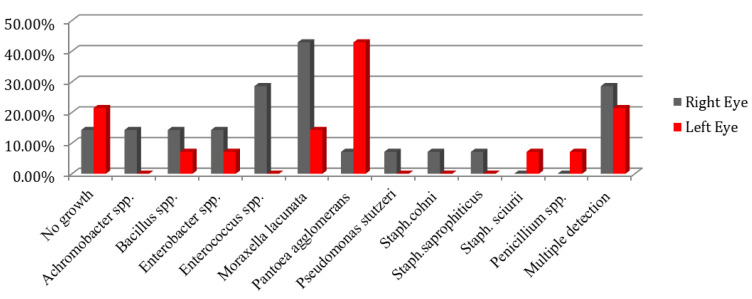
Percentage of conjunctival normal bacterial flora of right and left eye in healthy donkeys.

Of the 28 samples collected from 14 donkeys in our study, 21 swabs (75%) and 23 swabs (82.14%) were positive for bacteria cultivation in the direct culture and after multiplication, respectively. Only one eye (3.57%) was positive for fungi (*Penicillium* spp.). Bacterial growth was observed in 78.57% of samples from the left eye and in 85.71% from the right eye. Ten different types of bacteria were identified, along with one species of fungi (*Penicillium* spp.). More than one bacterial species has been isolated in seven (50%) animals in what was described as multiple species detection, whereas in six (42.85%) animals, the presence of the two bacterial species in one eye was confirmed. Only one donkey showed three bacterial species in the conjunctiva. *Pantoea agglomerans* was the most detected species, followed by *Moraxella lacunata*. Equally detected in healthy donkeys were *Pseudomonas stutzeri*, *Staphylococcus cohnii *subsp.* cohnii, Staphylococcus saprophiticus *subsp.* saprophiticus* and *Staphylococcus sciuri *subsp.* sciuri.* Table 1 shows the profile of detected bacterial species. No convergence of obtained isolates between the right and left eye of donkeys was observed in any individuals (Figure 1). Table 2 presents the susceptibility of identified *Staphylococci* based on the CLSI classification guidelines [14]. All three strains belonging to staphylococci were resistant to novobiocin.

## 4. Discussion

Among Equidae, from a clinical point of view, donkeys can be considered as more complicated ophthalmic patients than horses. The eyeball in donkeys is usually placed deeper than that of horses and is surrounded by a thicker periocular hair, making it more difficult to find early and mild lesions [16]. Conjunctivitis, corneal wounds and keratitis in donkeys most often arise as a result of trauma or a foreign body [17]. Bacterial complications may also result from the type of environment in which they live, auto-damage or the presence of flies, although this is more important in the case of working donkeys [17]. Therefore, knowledge of the commensal bacterial flora of a donkey’s conjunctival sac is applicable for the clinical diagnosis of primary or secondary bacterial pathological conditions, mainly in the case of chronic, recurrent and non-responsive antimicrobial treatment conjunctivitis [16].

In our study, bacteriological pathogen growth was detected in 82.14% of the swabs. Slightly more (86.9%) positive cultures of swabs taken from the conjunctival sac were obtained by Foti et al. in donkeys bred in Sicily (Italy) [7]. In turn, the percentage of positive bacterial cultures from donkeys described in this study is similar to data reported for horses examined in Lower Silesia in Poland (81%) [12].

A major part of the isolates were Gram-negative bacteria with a significant predominance of such species as *Pantoea agglomerans* and *Moraxella lacunata.* In the study conducted by Foti et al. in donkeys from Sicily (Italy), isolation of Gram-positive bacteria was higher, which may reflect geographical, environmental or seasonal differences [7]. In our study, we have also found bacteria from the genus *Staphylococcus* spp. in conjunctival swabs; however, they belonged to other species–*S. cohnii *subsp.* cohnii*, *S. saprophyticus *subsp.* saprophyticus* and *S. sciuri *subsp.* sciuri*—all coagulase-negative staphylococci (CoNS) and sensitive to methicillin. In the case of *Enterobacter* spp., we have obtained similar results: bacteria of this genus colonized the conjunctival sac of about 14% of donkeys.

Our findings differ from results obtained in healthy horses bred in Poland, which showed more frequent Gram-positive bacterial colonization of the conjunctival sac than Gram-negative. *Staphylococcus* spp. was the most frequently isolated genera among Gram-positive bacteria. Alike in our results for donkeys, there was no methicillin-resistant staphylococcus in equine conjunctival swabs. On the other hand, among Gram-negative bacteria, the dominant species in horses was *Moraxella lacunata* (11.6% of total isolates) [12]. *Moraxella lacunata* was abundantly isolated in donkeys in the following study. The remaining species of bacteria isolated from horses were different from those found in donkeys. Since both the horses described above and the donkeys from our study come from the same region, differences in the microorganisms found within the conjunctival sac are most likely to be a result of divergences in animal maintenance or the higher affinity of certain genera of bacteria for specific species of animals.

Among the obtained isolates, the most common, *Pantoea agglomerans*, seems to be interesting. It is an environmental bacterium that in humans was described as an opportunistic pathogen, an infectious agent that appears when the wound is contaminated with plant material or as a nosocomial infection [18]. Due to the fact that the donkeys participating in the study have never been in an equine hospital, it may be suspected that the presence of *P. agglomerans* on the mucous membranes of the conjunctival sac is associated with frequent contact of the eye with grass in the pasture. *P. agglomerans*, previously known as *Bacillus agglomerans* and *Enterobacter agglomerans,* is a Gram-negative aerobic bacillus that is omnipresent in nature [19]. In equines, *P. agglomerans* has been isolated as a potential causative pathogen in abortions in both horses and donkeys [20]. *P. agglomerans* was once isolated in studies of bacterial complicating factors in keratitis in horses and cattle with infectious keratoconjunctivitis [1,21]. In humans, in an ophthalmic context, it has been described as a pathogen causing endophthalmitis in two patients who developed penetrating trauma by rocks or with the tree branch [19]. Based on this correlation, in the case of donkeys, due to the high risk of damage to the eyeball by plant elements while in the pasture, it seems necessary to have a daily inspection by the owner and prompt treatment in the event of an injury in order to reduce the possibility of bacterial complications. Coagulase-negative *Staphylococcus* (CoNS) can pose a health risk to animals. Among Equidae, CoNS causes respiratory, ocular and uterine infections [22]. In addition, it has been found that staphylococci can cause nosocomial infections in horses in veterinary hospitals, mainly as postoperative infections, and may contribute to the need for intensive postoperative care, prolonged hospitalization, increased costs and mortality [22]. Therefore, the carriage of CoNS in donkeys should be taken into consideration in the diagnosis of ocular problems in this species.

In our study, no methicillin-resistant (MR) staphylococci were isolated in the conjunctival sac of donkeys. Currently, the resistance of staphylococci to antibiotics is a serious problem on a global scale [23]. Based on the literature, MR staphylococci have also been isolated from clinically healthy donkeys and horses and can pose a threat both to immunosuppressed animals and to people who come into contact with these animals [22,24,25].

The digestive tract of animals is a large reservoir of bacteria from the genus Enterococcus. In equine feces, enterococci such as *E. faecium*, *E. mundtii*, *E. faecalis E. hirae*, *E. gallinarum* and *E. casseliflavus* were found [26]. *E. asini* was only isolated from donkeys [27]. As in humans, enterococci can be opportunistic pathogens infecting animals [28]. The presence of this type of bacteria in the conjunctival sac of healthy individuals may be a result of contact with feces, for example, in pastures.

The possibility of microbial exchange between animals and humans is an important clinical issue. Donkeys are used in an array of areas of food production (e.g., milk, meat) and rehabilitation or recreational activities. The surrounding environment, such as humans or other animals, can be a source of microbes transmitted to animals. There are many cases of reverse zoonoses described in the literature [29]. Animals can be infected by humans with all types of microbes–bacteria, viruses, fungi and parasites [29]. In the case of bacteria, species such as methicillin-resistant *Staphylococcus aureus* (MRSA), *Mycobacterium tuberculosis* and *M. bovis*, *Streptococcus pneumoniae* and *Escherichia coli*, among others, have been observed to be able to cause reverse zoonosis [30,31,32,33]. Literature focuses mainly on cases of transmission of pathogenic bacteria to animals. For this reason, the microbes that inhabit these animals can pose a zoonotic risk to humans [34]. In the study conducted by Moodley et al., the same strains of *Staphylococcus* spp. were isolated from staff members, multiple horses and environmental sites within a veterinary hospital and farm [22]. The results indicated that there is a risk of bacteria transmission between Equidae and humans, either directly or through contaminated devices and the environment. Nevertheless, opportunistic microorganisms can also be passed from humans to animals or from animals to humans and cause infections if their immunity is weakened.

Although the studies on the microbiome are currently a very popular and innovative trend, helpful in the statement of the new status quo in the estimation of microbiota composition, still it is not a routine method used in the laboratory, especially in clinical cases. It is caused by the very high costs of the analysis, the inability to fully compare the results between different studies, as well as a limitation in the species-specific identification of microorganisms. Admittedly, such a study may confirm the presence of the non-cultivated in normal condition bacteria, but they have not yet replaced the traditional bacteriology, useful mainly for field doctors in the area of equine medicine.

## 5. Conclusions

In conclusion, our study confirmed that bacteria colonizing the conjunctival sac of donkeys under physiological conditions could be both commensal and conditionally pathogenic. Their occurrence may depend on environmental conditions, which was confirmed by the discrepancy between the obtained results and the results of studies conducted in Italy. The achieved conclusions also showed disparities in the composition of the conjunctival sac flora of donkeys and horses, despite the geographical proximity of both studies. Knowledge of the commensal conjunctival flora of donkeys is of great clinical importance by reason of greater exposure to corneal damage and infections than horses.

## Figures and Tables

**Table 1:** The profile of detected bacterial species in healthy donkey’s conjunctiva.

	Number/Swabs	Detected Species per Eye	Percentage of Affected Animals
*Achromobacter* spp.	2/28	7.14%	14.28% (2/14)
*Bacillus* spp.	3/28	10.71%	21.42% (3/14)
*Enterobacter* spp.	3/28	10.71%	14.28% (2/14)
*Enterococcus* spp.	4/28	14.28%	28.57% (4/14)
*Moraxella lacunata*	6/28	21.42%	42.85% (6/14)
*Pantoea agglomerans*	7/28	25%	50% (7/14)
*Pseudomonas stutzeri*	1/28	3.57%	7.14% (1/14)
*Staphylococcus cohnii *subsp.*cohnii*	1/28	3.57%	7.14% (1/14)
*Staphylococcus saprophyticus *subsp.*saprophyticus MSCNS*	1/28	3.57%	7.14 % (1/14)
*Staphylococcus sciuri *subsp.*sciuri MSCNS*	1/28	3.57%	7.14% (1/14)
Multiple detection	7/28	25%	50%

**Table 2:** Susceptibility of identified *Staphylococci* to Novobiocin, Polymyxin B and Cefoxitin according to the NCCLS standards; S–susceptible strain, R–resistant strain.

	Cefoxitin 30 µg (FOX 30)	Novobiocin 5 µg (NV 5)	Polymyxin B 300 µg (BP 300)
*Staphylococcus cohnii *subsp.*cohnii*	S	R	R
*Staphylococcus sciuri *subsp.*sciuri*	S	R	S
*Staphylococcus saprophyticus *subsp.* saprophyticus*	S	R	R

## Data Availability

Raw data for calculation of tables and figures are available from the corresponding author upon request.
